# The Relationship between Platelet Indices and Ultrasound, Clinical, Laboratory Parameters of Disease Activity in Patients with Rheumatoid Arthritis

**DOI:** 10.3390/jcm10225259

**Published:** 2021-11-12

**Authors:** Bożena Targońska-Stępniak, Krzysztof Grzechnik, Robert Zwolak

**Affiliations:** 1 Department of Rheumatology and Connective Tissue Diseases, Medical University of Lublin, 20-059 Lublin, Poland; 2 Department of Rheumatology and Connective Tissue Diseases, Independent Public Teaching Hospital No. 4, 20-954 Lublin, Poland; krzysiek.grzechink@gmail.com; 3 Department of Immunology, Center of Oncology of the Lublin Region St. Jana z Dukli, 20-090 Lublin, Poland; zwolakr@wp.pl

**Keywords:** platelet indices, platelet count, PDW, PCT, MPV, rheumatoid arthritis, ultrasound, disease activity

## Abstract

(1) Background: A proper assessment of disease activity is crucial for the management of a patient with rheumatoid arthritis (RA). Platelets seem to be involved in joint inflammation pathophysiology. Platelet indices (PIs) are markers of platelet activation, and include platelet count (PC), mean platelet volume (MPV), platelet distribution width (PDW) and plateletcrit (PCT). The purpose of the study was to assess the relationship between PIs and disease activity markers, both systemic (clinical, laboratory) and local (ultrasound, US), in patients with RA; (2) Methods: The study group consisted of 131 consecutive RA patients. The following assessments were performed: joint counts, Disease Activity Score (DAS28), complete blood cell counts, erythrocyte sedimentation rate (ESR), C-reactive protein (CRP), and US of 24 small joints; (3) Results: Mean values of PIs remained within the normal reference ranges. Values of PC, PCT, PDW were significantly associated with disease activity markers, both clinical (DAS28, joint counts) and laboratory (CRP, ESR). In patients with high disease activity, PC, PCT were significantly higher and PDW lower. PC was positively correlated with Power Doppler US (PDUS) score. In patients with features of RA severity (antibodies positivity, extra-articular manifestations) PC and PCT were positively associated with all US parameters (Grey Scale US, PDUS, Global scores); (4) Conclusions: In patients with RA, PC and PCT may serve as positive disease activity markers and PDW may serve as a negative marker. PIs may be used as reliable, inexpensive markers of RA systemic activity; they may also serve as markers of local inflammation in the joints affected by RA.

## 1. Introduction

Rheumatoid arthritis (RA) is a chronic autoimmune disease, characterized by inflammation and progressive destruction of synovial joints. An assessment of RA activity is conducted by measuring inflammatory markers, which closely correlate with the clinical disease activity. These include erythrocyte sedimentation rate (ESR) and C-reactive protein (CRP), which are widely used in daily practice [1,2]. The clinical disease activity in patients with RA is most commonly assessed by the Disease Activity Score of 28 joints (DAS28), which is calculated by the number of tender joints, swollen joints, patient global assessment, and the ESR value or CRP concentration. The value of DAS28 substantially relies on subjective assessments of a patient and/or physician [1,3]. In recent decades, ultrasound (US) imaging of joints has been introduced into the diagnostic and therapeutic process. A US examination may verify the clinical assessment of affected joints and improve the management of patients with RA [4,5].

A growing body of evidence suggest that platelets are involved in inflammation, in addition to their crucial role in hemostasis and thrombosis [6,7]. Systemic rheumatoid inflammation mediated by numerous cytokines, growth factors and autoantibodies, stimulates platelet production in the bone marrow. Platelets are anucleate cells, but can synthesize proteins on their mRNA and can produce microparticles (MPs) [6]. A high number of platelets and substantial number of MPs are detectable within RA synovium and synovial fluid [6].

Platelet indices (PIs) are markers of platelet activation, parameters that could easily be obtained as a part of an automatic blood count [1,8]. They include platelet count (PC), mean platelet volume (MPV), platelet distribution width (PDW) and plateletcrit (PCT). MPV signifies the average size of platelets in the blood. PCT measures total platelet mass as a percentage of volume occupied in the blood. PDW is a marker of platelet anisocytosis, describes the size distribution of the platelets produced by megakaryocytes [8].

Recently, PIs have been thoroughly investigated as novel biomarkers in both acute and chronic diseases [8]. Several studies confirmed the relevance of PI changes in the course of diabetes mellitus (DM) [8,9,10,11], cardiovascular disease (CVD) [8,12,13], cancers [8,9,13], acute surgical conditions [8,14].

The relationship has been also reported between PIs and disease activity in patients with RA. However, the literature data on this association are inconsistent and even controversial [1,2,15,16,17,18,19,20,21,22,23]. In patients with active RA, MPV value was significantly lower [2,15,16,17,18] and increased after treatment [2,15,19]. On the contrary, in another study, PC and MPV values were significantly higher in RA patients and positively correlated with DAS28. Moreover, PIs decreased after RA therapy [20]. A positive correlation was found between DAS28 and both PC and PDW [1]. It was also found that, in patients with RA, MPV was significantly higher and PDW was lower when compared with controls. Negative correlations were reported between DAS28-CRP and MPV, PDW [21]. PCT was found to be a positive acute phase reactant in patients with RA, while MPV and PDW were negative acute phase markers [22]. The recent meta-analysis reported that PC was significantly higher in patients with RA than in controls, while MPV, and PDW values were non-significantly different [23].

The purpose of this study was to assess the relationships between PIs (PC, MPV, PCT, PDW) and the disease activity markers, both systemic (clinical, laboratory) and local (US parameters) in patients with RA.

## 2. Materials and Methods

### 2.1. Study Population

The study group consisted of 131 RA patients, hospitalized in the Department of Rheumatology and Connective Tissue Diseases, Medical University of Lublin. All patients fulfilled the American College of Rheumatology (ACR)/European League Against Rheumatism (EULAR) classification criteria for RA [24]. This study was conducted in accordance with the Declaration of Helsinki of 1975, revised in 2013. The study design was approved by the Ethics Committee of the Medical University of Lublin (approval number KE-0254/319/2016, obtained before undertaking the research). Informed consent was obtained from each patient after an adequate explanation of the study design, prior to their inclusion in the study.

### 2.2. Clinical and Laboratory Findings

Baseline demographics and clinical data were collected through medical interviews and a review of their medical history and records. Data, including the age of the disease onset, RA duration, IgM rheumatoid factor (RF-IgM) and anti-citrullinated peptide antibodies (anti-CCP) positivity, and extra-articular manifestations during the course of the disease, were retrieved.

A physical examination was performed, including tender joint count (TJC) and swollen joint count (SJC). The disease activity of RA was determined using the 28 joints Disease Activity Score system (DAS28), calculated with TJC, SJC, ESR, and patient global assessment (PGA) in visual analogue scale (VAS) [3]. The cut point for low disease activity was DAS28 value ≤ 3.2, and for high disease activity, it was >5.1. The ability to perform daily activities was assessed using a modified Health Assessment Questionnaire (M-HAQ), with a range 0–3 (with score 0 representing no impairment of function) [25]. The erosive form of RA was identified in patients who had erosions on joint surfaces of bones in X-rays of hands and/or feet, by a trained radiologist [26].

Blood was collected after overnight fasting. Blood tests in all patients included ESR, CRP, complete blood cell count (CBC), including hemoglobin (Hb), white blood cell count (WBC), PC, MPV, PCT, PDW. All samples were analyzed with ADVIA 2120i System automated cell counter (Siemens-Germany). The normal ranges for PIs were as follows: PC 150–400 × 10^9^/L; MPV 7.4–10.4 fl; PCT 0.12–0.3%; PDW 40.0–60.0%.

### 2.3. Ultrasound Imaging of Joints

Ultrasound (US) imaging was performed, including 24 bilateral joints: wrists (radio-carpal, midcarpal), metacarpophalangeal (MCP), hand proximal interphalangeal (PIP), thumb interphalangeal (IP), and fifth metatarsophalangeal (MTP) joints. The US examination was performed using a machine (MyLab25 Gold, Esaote, Genova, Italy) equipped with an 18 MHz broadband high-frequency linear array transducer for synovium hypertrophy and effusion (grey scale ultrasound, GSUS), with identical Power Doppler settings (pulse repetition frequency (PRF)—700 Hz; gain was increased to the maximum level, not generating random noise with the lowest wall filters).

The most commonly used scoring system was applied to assess synovitis:

1. Semi-quantitative grey scale (GS) for grading synovial hypertrophy (0–3) in each joint:
  - Grade 0: normal joint with no synovial hypertrophy;
  - Grade 1: synovial hypertrophy up to the level of the horizontal line connecting the bone surfaces of an examined joint;
  - Grade 2: synovial hypertrophy extending beyond the joint line but with the upper surface flat to the underlying bones;
  - Grade 3: synovial hypertrophy extending beyond the joint line but with the upper surface convex to the underlying bones.
2. Power Doppler ultrasound (PDUS) semi-quantitative scale (0–3) in each joint:
  - Grade 0: no Doppler activity;
  - Grade 1: up to three single Doppler spots, or up to one confluent spot and two single spots, or up to two confluent spots;
  - Grade 2: greater than grade 1 but <50% Doppler signals in the total GS background;
  - Grade 3: greater than grade 2 and >50% Doppler signals of the GS background [27].

Images were obtained according to the EULAR recommendations with a longitudinal scan, using either a dorsal view (wrists), a dorsal or volar (plantar) view (MCPs, PIPs, thumb Ips, and fifth MTPs) for both GSUS and PDUS scores [28]. The highest score from the dorsal or volar (plantar) view of MCPs, PIPs, thumb IPs, and fifth MTPs was taken for calculation. Then, we calculated the GSUS and PDUS scores by summing the scores obtained as a result of an assessment of individual joints (range 0–72). The global score was calculated by summing GSUS and PDUS scores from all examined joints (range 0–144) [29].

### 2.4. Statistical Analysis

Continuous variables are presented using the mean ± standard deviation (SD) or median and interquartile range (IQR) if the data were parametric or nonparametric, respectively. Categorical data were summarized as absolute numbers and percentages. The results were tested for normality using Kolmogorov–Smirnov’s test. To compare continuous variables in subgroups of patients, Student’s *t*-test or the nonparametric Mann–Whitney U test, were used. Correlations between the quantitative variables were assessed by Spearman’s or Pearson’s correlation test. A multiple linear regression test was performed, introducing variables that showed a statistically significant association with certain parameters. For all tests, *p* values < 0.05 were considered significant. All statistical analyses were performed using the StatSoft STATISTICA 12 application.

## 3. Results

### 3.1. Demographic and Disease-Related Variables in RA Patients

The study group mainly consisted of women (almost 80%). The disease duration >10 years was found in over 55% of patients. The vast majority of patients had an erosive form of RA, and over 80% were seropositive (RF-IgM and/or anti-CCP). Extra-articular symptoms (rheumatoid nodules, sicca syndrome, interstitial lung disease, and vasculitis) in the course of the disease were observed in over 50% of patients (Table 1).

At the time of examination, conventional synthetic disease modifying anti-rheumatic drugs (csDMARDs) were used in 128 patients and included methotrexate (MTX) in 119 (90.8%) patients (dose 10–25 mg/week, in monotherapy or combination), leflunomide (LEF) 14 (10.7%), hydroxychloroquine (HCQ) or chloroquine (CQ) 51 (38.9%), sulfasalazine (SS) 13 (9.9%) and cyclosporine 1 (0.8%). Low-dose glucocorticoid (GC) therapy (prednisone ≤ 10 mg/day) was used in 86 (65.6%) patients (Table 1).

High disease activity (DAS28 > 5.1) at the time of examination was found in about 1/3 of patients (Table 2). The mean values of PC, PCT, MPV and PDW remained within the normal reference ranges.

### 3.2. Comparison of PIs in Certain Groups of Patients with RA

**Figure 1:**
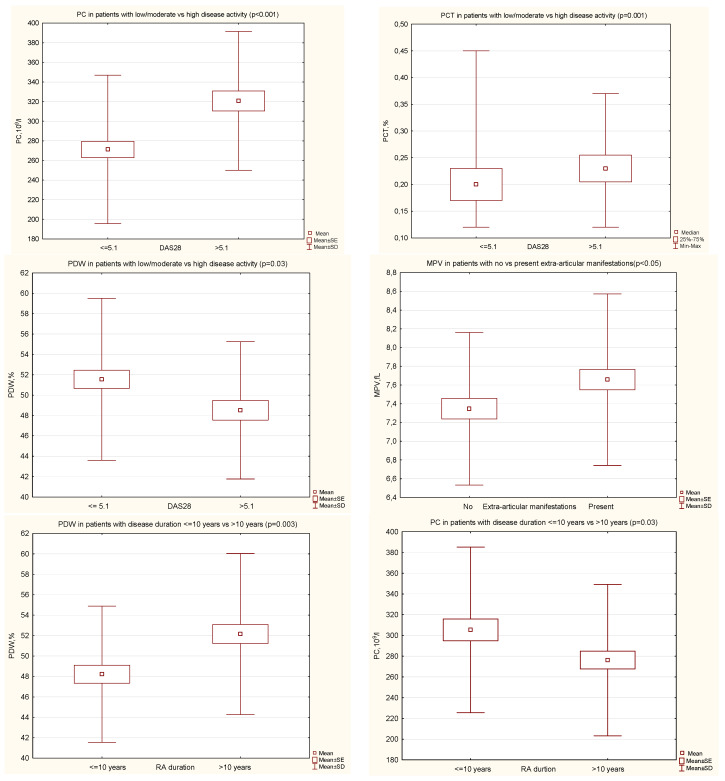
Significant differences of PIs in certain groups of patients with RA. DAS28, disease activity score in 28 joints; MPV, mean platelet volume; PC, platelet count; PCT, plateletcrit; PDW, platelet distribution width; RA, rheumatoid arthritis.

In RA patients with high disease activity (DAS28 > 5.1), when compared with those with moderate or low disease activity (DAS28 ≤ 5.1), we found significantly higher mean values of PC (320.7 ± 70.7 vs. 271.3 ± 75.6, *p* < 0.001) and PCT (0.23 (0.21–0.26) vs. 0.21 (0.17–0.23), *p* = 0.001), and the mean value of PDW was significantly lower (48.5 ± 6.7 vs. 51.6 ± 7.9, *p* = 0.03) (Figure 1). The MPV value was comparable in both groups.

The mean value of MPV was significantly higher in patients with extra-articular manifestations during the course of RA, as compared with no extra-articular symptoms (7.7 ± 0.9 vs. 7.3 ± 0.8, *p* < 0.05) (Figure 1). The PC, PCT, PDW values were comparable in both groups.

In patients with a long RA duration (>10 years), as compared with a shorter disease duration, the mean value of PDW was significantly higher (52.2 ± 7.9 vs. 48.2 ± 6.7, *p* = 0.003) and the mean value of PC significantly lower (276.27 ± 73.0 vs. 305.4 ± 79.8, *p* = 0.03) (Figure 1). The MPV and PCT values were comparable in both groups. The disease activity assessed by DAS28 was comparable in both groups.

### 3.3. The Relationship between PIs (PC, MPV, PCT, PDW) and the Clinical Disease Activity Markers, and the Disease Duration, and Treatment Used

Positive correlations were found between PC and almost all the clinical disease activity parameters, as well as a negative correlation with disease duration. Positive correlations were also found between PCT and almost all the clinical disease activity parameters, except M-HAQ (Table 3).

Negative correlations were noted between PDW and DAS28, SJC, and a positive correlation was noted with disease duration. There was a negative correlation between MPV and PGA and a positive one between MPV and disease duration (Table 3).

In the multiple linear regression analysis, significantly positive associations were confirmed for PC with DAS28 (b = 0.49, *p* = 0.02); MPV with the disease duration (b = 0.21, *p* = 0.02); as well as positive association for PCT with DAS28 (b = 0.57, *p* = 0.01) and negative with PGA (b = −0.41, *p* = 0.03).

The mean value of PDW was significantly higher in patients that were currently treated vs. not treated with biological DMARDs (52.1 ± 8.1 vs. 48.8 ± 6.8, *p* = 0.02). There were no other significant differences in PIs in patients treated with synthetic DMARDs or GC.

### 3.4. The Relationship between PIs (PC, MPV, PCT, PDW) and Laboratory Disease Activity Markers

Positive correlations were found between PC, PCT and all the laboratory disease activity parameters. Negative correlations were noted between PDW and CRP, ESR (Table 3).

In the multiple linear regression analysis, significant positive associations were confirmed for PC with ESR (b = 0.24, *p* = 0.02), WBC (b = 0.26, *p* = 0.003) and negative association with Hb (b = −0.20, *p* = 0.03); positive association for PCT with WBC (b = 0.24, *p* = 0.006) and negative with Hb (b= −0.24, *p* = 0.01); negative association for PDW with ESR (b = −0.28, *p* = 0.004).

### 3.5. The Relationship between PIs and US Parameters in the Group of 131 Patients with RA

**Figure 2:**
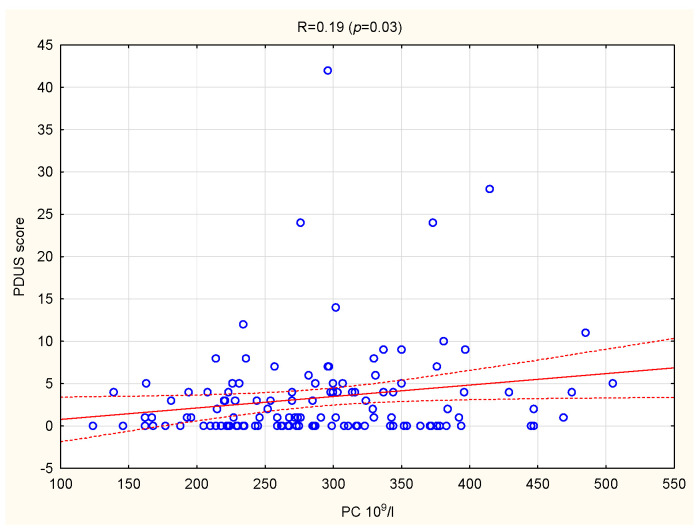
Correlation between PC and PDUS score in patients with RA. PC, platelet count; PDUS, power doppler ultrasound; RA, rheumatoid arthritis.

In the whole group of patients, a positive association was confirmed between PC and PDUS (R = 0.19, *p* = 0.03) (Figure 2). There were no other significant correlations between PIs and US parameters.

### 3.6. The Relationship between PIs and US Parameters in Certain Groups of Patients with RA

The significant positive correlations were found between PIs (PC, PCT) and US parameters, in certain groups of patients: anti-CCP positive, RF-IgM positive, with extra-articular manifestations (Table 4). In patients with the disease duration < 10 years, a negative correlation was observed between PDW and US parameters (Table 4). No significant correlations were observed between MPV and US parameters.

## 4. Discussion

Our study showed that, in patients with RA, platelet indices (PIs) were significantly associated with both clinical (DAS28, TJC, SJC, PGA, morning stiffness) and laboratory (CRP, ESR, WBC, Hb) disease activity markers.

In this study, we found a significant positive correlation between PC and PDUS. We observed that PIs (PC, PCT) were positively correlated with US parameters (GSUS, PDUS, Global score) in patients with symptoms associated with the severity of RA (anti-CCP and RF-IgM positivity, extra-articular manifestations). In patients with a shorter disease duration (up to 10 years), PDW was inversely associated with GSUS and PDUS scores. To the best of our knowledge, this is the first study that investigated the relationships between PIs and US disease activity parameters in patients with RA. The results demonstrated the significant role of platelets in local inflammation of joints.

The US imaging of joints is widely used to evaluate inflammatory arthritis, because it visualizes the extent and severity of local inflammation of joints. According to many studies, in patients with RA, PDUS score correlated with serum inflammatory markers and SJC; GSUS score correlated better with physical examination (TJC, SJC); while the Global score was correlated with DAS28 and useful in the assessment of overall disease activity [5,30]. In this study, we found that PDUS, which confirms neoangiogenesis and active synovitis, was significantly associated with PC.

In the literature, it was demonstrated that platelets are directly involved in synovial inflammation and cartilage destruction. It has been suggested that platelet activation can be mediated by anti-citrullinated protein antibodies (ACPA). Markers of platelet activation correlate with high ACPA titers [6]. In inflammatory arthritis, platelet counts in synovial fluid are associated with RF and markers of synovial leucocyte activation [7].

In this study, the mean values of PIs remained within the normal reference ranges in the whole group of patients with RA. However, PIs differed significantly in certain groups of patients. PC and PCT were higher, and PDW lower, in patients with high disease activity, which is a poor prognostic factor according to the EULAR recommendations [31], when compared to patients with low/moderate disease activity.

The values of PC and PCT were positively associated with all clinical and laboratory parameters as well as PC with US parameters, and may serve as positive disease activity markers. PDW was inversely associated with clinical and laboratory parameters (DAS28, SJC, CRP, ESR) and may serve as a negative disease activity marker. We found no significant association between MPV and both clinical and laboratory disease activity markers. MPV was only higher in patients with extra-articular manifestations, as compared with no extra-articular symptoms in the course of RA.

We observed significant correlations between PIs and disease duration (positive with PDW and MPV, negative with PC), but no correlation with the age of patients. In patients with a longer RA duration (>10 years) as compared with a shorter disease duration, PDW was significantly higher, and PC was lower.

Our results are consistent with the data in the literature. Positive correlations were reported between PC and DAS28 in patients with RA [1]. Nevertheless, a positive correlation was also detected between PDW and DAS28, and no correlation was detected with PCT. The mean value of PDW was much lower in that study as compared with our study [1]. The results of the recent meta-analysis demonstrated that PC was significantly higher in RA patients than in controls, was strongly correlated with ESR and weakly correlated with disease activity score. MPV and PDW presented nonsignificant differences between patients and controls [23].

It is reported that PCT may have a screening role in the detection platelet quantitative abnormalities. It is nonlinearly correlated with PC and indicates a comparable clinical implication. In patients with RA, PC and PCT values were correlated with DAS28 and inflammatory markers [17]. PCT was found to be a positive acute phase reactant in patients with active RA [22].

In the literature, a low PDW value, below the normal range, was observed in non-malignant tumors. A high PDW value was reported in DM-related retinopathy and nephropathy, acute cholelithiasis, myocardial infarction as a result of platelet activation [8,10]. In patients with RA, no significant association was observed between PDW value and DAS28 score [16,17]; there was no correlation with inflammatory markers [17]. In another study, PDW was decreased in high-disease-activity RA patients, when compared with controls [18,21]. PDW was found to be a negative acute phase reactant in patients with active RA [22].

MPV is considered as a marker of platelet activity and a potential biomarker of prognosis in different diseases (myocardial infarction, DM, pancreatic ductal adenocarcinoma [8,9,10]. It has been suggested that MPV may be associated with RA activity, although the data on this subject are inconsistent [15]. MPV was reported to be decreased in active RA [16,17] when compared with osteoarthritis group [2,15] and with normal individuals [2,18]. After treatment MPV values increased [2,19]. On the contrary, MPV value was reported to be higher in patients with RA, compared to a control group [20,21], and positively correlated with DAS28 and inflammatory parameters (CRP, ESR) [20]. MPV significantly decreased alongside PC, CRP, IL-6, in response to anti-TNF-alpha and conventional treatment [7,20]. PIs values may be related to the method of laboratory analysis. Many factors may modify MPV (race, age, smoking, alcohol consumption, physical activity) [8]. These could be the reason for the controversy among different studies.

It can be seen that PC is inversely associated with age and decreased PC seems to be a part of the biological aging process. A significant decrease was shown (10 × 10^3^ platelets/µL) in individuals of 60–69 years of age as compared with those between 20 and 59 years of age. This effect could be associated with a lower stem cell reserve in older individuals, or a reduced PC as a biological advantage, because individuals with a lower PC seem to have a better chance of reaching an older age [32].

Our study has some potential limitations. First, the relatively small number of patients included in the study; a higher number of patients could enable better statistical evaluation. Second, we evaluated the relationship of PIs with DAS28, and no other indices such as CDAI or SDAI. Third, we could not exclude the influence of other concurrent medical conditions or smoking, which could potentially influence the value of PIs. Fourth, we could not exclude the influence of concomitant treatment (DMARDs or GC), because the study was performed in real-life RA patients. Fifth, the study design was cross-sectional.

Our study also has several strengths. First, to the best of our knowledge, it is the first study assessing the associations between PIs and different US scores in addition to clinical and laboratory parameters. Second, it has detailed characteristics of the patients, which were considered in all aspects of RA pathology. Third, patients were not selected for the study—they are real patients. Fourth, the same experienced physician performed all the US measurements, thereby eliminating any interpersonal variations. Fifth, all the performed assessments are available on an outpatient basis.

## 5. Conclusions

In this study, we found that PIs correlate with parameters of RA activity, both laboratory (CRP and ESR) and clinical (DAS28, SJC, and TJC). The results of our study point towards a significant relationship between PIs and US disease activity parameters. PC and PCT may serve as a positive disease activity marker and PDW as a negative disease activity marker in patients with RA. PIs may be used as reliable markers of RA systemic activity; they may also serve as markers of local inflammation in the joints affected by RA.

## Figures and Tables

**Table 1:** Characteristics of patients with RA.

Data	Results (*n* = 131)
Age, years	54.0 (±11.9)
Gender, female/male (*n*, %)	104 (79.4)/27 (20.6)
Disease duration, years	14.2 (±10.9)
Disease duration > 10 years	73 (55.7)
Positive RF-IgM (*n*, %)	112 (85.5)
Positive anti-CCP (*n*, %)	108 (82.4)
Extra-articular manifestations (*n*, %)	71 (54.2)
Erosions (hands/feet) (*n*, %)	123 (93.9)
Current synthetic DMARD used (*n*, %)	128 (97.8)
MTX	119 (90.8)
Anti-malarial drug	51 (38.9)
Leflunomide	14 (10.7)
Other	14 (10.7)
Current biological DMARD used (*n*, %)	64 (48.9)
Anti-TNF	31 (23.7)
Other	33 (25.2)
Current low dose glucocorticoid use (*n*, %)	86 (65.6)

Values are displayed as mean ± standard deviation (SD), median (IQR) or frequencies with corresponding percentages (%). anti-CCP, anti-cyclic citrullinated protein antibodies; anti-TNF, anti-tumor necrosis factor; DMARD, diseases modifying anti-rheumatic drug; GC, glucocorticosteroid; MTX, methotrexate; RA, rheumatoid arthritis; RF-IgM, IgM rheumatoid factor.

**Table 2:** Laboratory, clinical and US parameters in RA patients.

Data	Results (*n* = 131)
Laboratory results:	
Hemoglobin, g/dL	12.8 (±1.4)
WBC, 10^9^/L	7.0 (±2.7)
PC,10^9^/L	289.1 (±77.2)
PCT, %	0.21 (0.18–0.24)
MPV, fl	7.5 (±0.9)
PDW, %	50.4 (±7.6)
CRP, mg/L	9.3 (2.0–19.0)
ESR, mm/h	27 (14–51)
Clinical parameters of RA activity:	
TJC	4 (1–10)
SJC	2 (0–6)
PGA (VAS), mm	38.2 (±26.0)
Morning stiffness, minutes	30.0 (10–75)
DAS28	4.24 (±1.8)
High Disease Activity (DAS28 > 5.1) (*n*, %)	48 (36.6)
Remission/Low Disease Activity (DAS28 < 3.2) (*n*, %)	46 (35.1)
M-HAQ	1.4 (±0.8)
US parameters of RA activity:	
GSUS score (hypertrophy)	9 (3–19)
PDUS score	1 (0–4)
Global score	10.5 (4–23)
Global score = 0 (*n*, %)	15 (12.1)

Values are displayed as mean ± standard deviation (SD), median (IQR) or frequencies with corresponding percentages (%); CRP, C-reactive protein; DAS28, disease activity score in 28 joints; ESR, erythrocyte sedimentation rate; GSUS, Grey Scale Ultrasound; PGA, patient global assessment; M-HAQ–modified health assessment questionnaire; MPV, mean platelet volume; PC, platelet count; PCT, plateletcrit; PDUS, power doppler ultrasound; PDW, platelet distribution width; RA, rheumatoid arthritis; SJC, swollen joints count TJC, tender joint count; VAS, Visual Analogue Scale, WBC, white blood cell count.

**Table 3:** Correlations between clinical and laboratory parameters and PIs in patients with RA.

Data/*p* Value/R	PC	PCT	MPV	PDW
DAS28	<0.001	<0.001	NS	0.02
	0.42	0.32		−0.22
TJC	<0.001	0.01	NS	NS
	0.29	0.23		
SJC	<0.001	0.002	NS	0.008
	0.34	0.27		−0.23
PGA	0.005	0.02	0.02	NS
	0.26	0.22	−0.21	
Morning stiffness	<0.001	<0.001	NS	NS
	0.33	0.32		
M-HAQ	0.05	NS	NS	NS
	0.19			
Disease duration	0.008	NS	0.02	0.02
	−0.23		0.21	0.21
CRP	<0.001	<0.001	NS	0.03
	0.35	0.33		−0.2
ESR	<0.001	<0.001	NS	<0.001
	0.43	0.42		−0.33
WBC	0.002	0.004	NS	NS
	0.29	0.25		
Hb	0.02	<0.001	NS	NS
	−0.22	−0.29		

CRP, C-reactive protein; DAS28, disease activity score in 28 joints; ESR, erythrocyte sedimentation rate; Hb, hemoglobin; M-HAQ, modified health assessment questionnaire; PGA, patient global assessment; SJC, swollen joints count TJC, tender joint count; VAS, Visual Analogue Scale; WBC, white blood cell count.

**Table 4:** Significant correlations between PIs and US parameters in certain groups of patients with RA.

Data/*p* Value/R	GSUS Score	PDUS Score	Global Score
Patients anti-CCP positive:			
PC	NS	0.02	NS
		0.22	
PCT	NS	0.03	NS
		0.22	
Patients RF-IgM positive:			
PC	0.04	0.001	0.02
	0.20	0.32	0.23
PCT	NS	0.009	0.04
		0.26	0.20
Patients with extra-articular symptoms:			
PC	0.002	<0.001	<0.001
	0.36	0.44	0.39
PCT	0.03	0.01	0.02
	0.32	0.30	0.28
Patients with RA duration < 10 years			
PDW	0.04	0.03	NS
	−0.28	−0.29	

anti-CCP, anti-cyclic citrullinated protein antibodies; GSUS, Grey Scale Ultrasound; PC, platelet count; PCT, plateletcrit; PDUS, power doppler ultrasound; PDW, platelet distribution width; RA, rheumatoid arthritis; RF-IgM, IgM rheumatoid factor.

## Data Availability

The data supporting these results are available from the corresponding author on reasonable request.
